# Horner Syndrome Secondary to Osteochondroma of the First Rib: A Case Report

**DOI:** 10.7759/cureus.14531

**Published:** 2021-04-17

**Authors:** Erika Tvedten, Jordan Richardson, Tarek Husien, Mohamed Zghouzi

**Affiliations:** 1 Internal Medicine, Detroit Medical Center (DMC), Detroit, USA; 2 Pediatrics, Detroit Medical Center (DMC) Children's Hospital of Michigan, Detroit, USA

**Keywords:** horner syndrome, osteochondroma, case report, radiology, neurosurgery, pediatric

## Abstract

Osteochondroma is the most common benign tumor of bone that often produces no symptoms unless the enlarged mass affects nearby structures. Rarely, Horner syndrome can be caused by an osteochondroma.

A five-year-old female with a past medical history of seizure-like activity presented to the emergency department on three separate occasions within one month. She exhibited neurological deficits, including miosis and ptosis, resulting in the diagnosis of Horner syndrome. Computerized tomography (CT) demonstrated a calcified and ossified lesion arising from the right first rib and transverse process that was suspicious for an osteochondroma or chondrosarcoma with neuroblastoma lower on the differential diagnosis. Given the patient's escalating clinical symptomatology and suspicious features of the lesion, a CT guided-bone biopsy was performed. Pathology revealed an osteochondroma that was eventually resected by neurologic and orthopedic surgeries.

In this case report, we review the sympathetic innervation to the head, eye, and neck, the most common etiologies of Horner syndrome, and elucidate imaging modalities useful for diagnosing osteochondroma. Horner syndrome secondary to osteochondroma of the first rib has been documented only once before.

## Introduction

Horner syndrome is a neurological disorder that presents with the classic triad of ptosis (drooping of the upper eyelid), miosis (a small pupil), and anhidrosis (absence of sweating) [1,2]. Horner syndrome's etiology is widespread as it can be due to a lesion anywhere along the path of sympathetic nervous system (SNS) fibers that innervate the head, eye, and neck. Rarely, Horner syndrome can be caused by an osteochondroma [3-8].

Osteochondroma is the most common benign tumor of bone with a prevalence of 0.44% that exhibits peak incidence in the second decade of life [9-11]. The tumor localizes to the growth plate of long bones, especially the distal femur and proximal tibia, and grows as a lateral projection [10]. Clinically, osteochondromas are usually asymptomatic, but occasionally, when they are symptomatic, they can be painful and palpable; treatment then consists of surgical removal of the tumor [9,10]. There is a one percent risk that the overlying cartilage in an osteochondroma transforms into malignant chondrosarcoma. Malignant progression can be gauged by the thickness of the cartilaginous cap [10,12]. Moreover, osteochondromas can be part of a condition called multiple hereditary exostoses (MHE), a bone disease characterized by several osteochondromas. MHE osteochondromas have a 5%-10% chance of becoming malignant [9,10].

This case represents a rare and unique presentation of Horner syndrome. A few other studies diagnosed Horner syndrome secondary to osteochondroma of the clavicle [3-6]. Another report identified Horner syndrome secondary to osteochondroma of the seventh cervical vertebra [7]. To our knowledge, there is only one other instance of Horner syndrome secondary to osteochondroma of the rib, and this was published in 1948 [8]. This case report illustrates the extreme difficulty in identifying osteochondromas radiologically. We discuss the various imaging modalities used to arrive at a diagnosis of osteochondroma of the rib. Lastly, osteochondromas have malignant potential, so it is crucial to accurately detect disease burden to determine plausible therapy candidates.

## Case presentation

**Figure 1:**
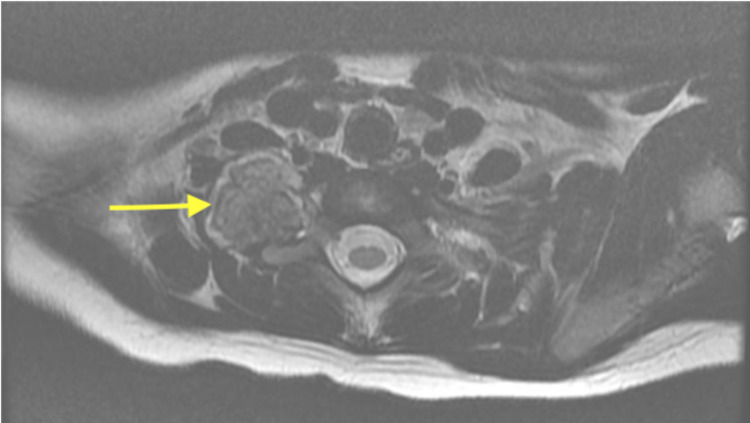
MRI of cervical spine (axial T2) revealing osteochondroma of the right first rib (at yellow arrowhead) MRI - Magnetic Resonance Imaging Right paraspinal mass that appears to arise from the right first rib with continuity of the medullary cavity and bright peripheral T2 signal suggesting a cartilage cap. Findings are most consistent with osteochondroma. However, given the widening of the adjacent neural foramina and apparent extension to the neural foramina, ganglioneuroblastoma could be considered, though this would be less likely given the profuse calcification.

A five-year-old female presented to the ED with left eye pain, gastrointestinal symptoms and fatigue. This was her third visit to the ED in the last month. The patient presented to the ED the first time for seizure-like activity and was discharged home to follow up with pediatric neurology. Shortly after this incident, her schoolteacher noticed a discrepancy between the size of the patient's pupils, which prompted a return to the ED where physicians witnessed ptosis and miosis of the right eye. A head CT was negative, and the patient was told to follow up with neurology as an outpatient. Upon the third and final visit, a magnetic resonance imaging (MRI) of the brain and cervical spine and a magnetic resonance angiography (MRA) head and neck were obtained. The cervical spine MRI revealed an enhancing mixed T2 hypointense mass with a peripheral T2 hyperintense rim representing the cartilaginous cap (Figure 1). It was suggested that a calcification within the inferior right paraspinal neck reached from the C7 to T1 levels with extension into the C6-T2 neural foramen without dural involvement. Further evaluation with CT thorax was recommended and the patient was admitted.

**Figure 2:**
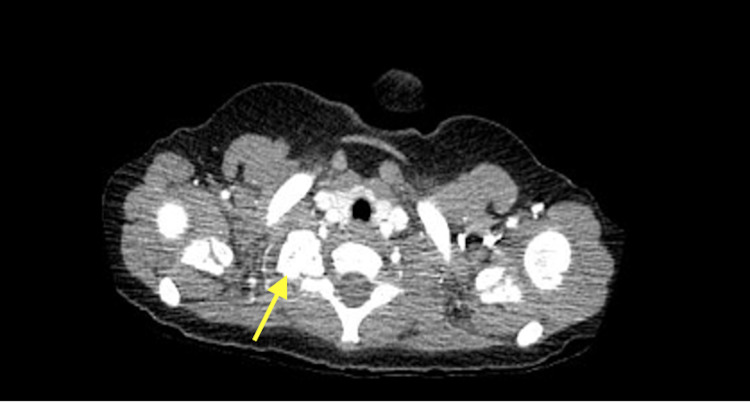
CT thorax with contrast showing osteochondroma (at yellow arrowhead) arising from the right first rib and possibly the transverse process of T1 1.8 x 1.8 x 1.6 cm calcified/ossified mass, appearing to arise from the right first rib, suggestive of osteochondroma. The almost complete calcification of this lesion makes the diagnosis of neuroblastoma less likely. CT - Computerized Tomography

**Figure 3:**
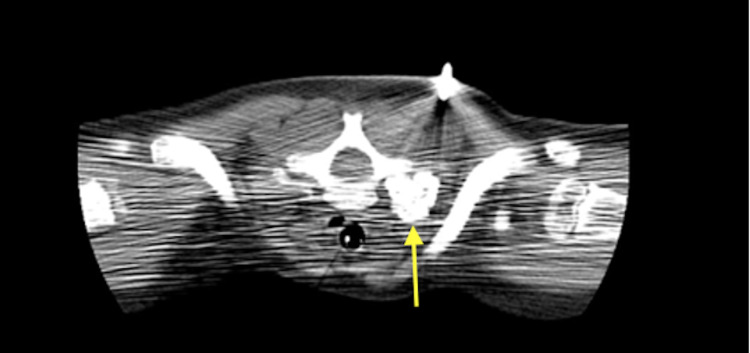
CT-guided bone biopsy of right cervicothoracic paraspinal mass (at yellow arrowhead) that revealed osteochondroma Patient was in a prone position for the procedure. CT - Computerized Tomography

Ophthalmology confirmed the diagnosis of Horner syndrome. A thoracic CT with contrast revealed a calcified mass arising from the right first rib or possibly the transverse process of T1 (Figure 2). The mass measured 1.8 x 1.8 x 1.6 cm (anterior/posterior x transverse x cranial/caudal, respectively), and no soft tissue components were identified. There were implications of thinning and remodeling of the underlying second rib; however, no osseous connection to the mass was seen. Since the patient remained at baseline activity, the primary team believed the rest of the workup could be done as an outpatient. A CT-guided bone biopsy took place, as illustrated in Figure 3. Multiple irregularly shaped, unoriented fragments of tan-brown, firm, bony tissue was collected weighing 2.4 g and aggregating to 2 x 2 x 0.9 cm. Gross examination revealed multiple fragments were surfaced by gray, smooth cartilage. Sectioning exhibited tan to red, hard bony tissue with gray, rubbery cartilaginous tissue measuring up to 0.3 cm in maximum thickness. Histological analysis revealed fragments of bone surfaced by a cartilaginous cap undergoing endochondral ossification to mature bone with hypocellular and normocellular bone marrow consistent with a diagnosis of osteochondroma.

Additionally, no evidence of malignancy was appreciated. Orthopedic surgery and neurosurgery collectively performed an excision of the right first rib osteochondroma, including a portion of the first rib and a partial hemilaminectomy and foraminotomies C6-7 and C7-T1. There were no acute complications with the surgery. The patient was neurologically stable post-surgery and cleared by physical therapy for discharge home.

## Discussion

Horner syndrome is caused by a lesion to the SNS fibers that supply structures in the body superior to the neck. First-order sympathetic fibers (central) originate in the hypothalamus, where they travel to the brainstem, cervical and thoracic spinal cord before reaching the ciliospinal center (C8-T2). From here, second-order SNS fibers (preganglionic) exit the spinal cord, pass over the pulmonary apex, travel through the stellate ganglion, and then synapse on the superior cervical ganglion. Lastly, third-order SNS fibers (postganglionic) travel along the internal carotid artery and ophthalmic nerve before eventually synapsing on the iris dilator muscle [1,2].

The SNS takes a long, tortuous path to the head, allowing us to categorize Horner syndrome lesions into central, preganglionic, and postganglionic. Common central causes of Horner syndrome include brainstem strokes (e.g. Wallenberg Syndrome), brain tumors, brainstem (pontine) hemorrhages, demyelinating diseases like multiple sclerosis, and Malformations (e.g. Arnold-Chiari) [1,2]. Preganglionic causes include Pancoast tumors of the lung, cervical ribs, iatrogenic causes (e.g. central venous catheterization, birth trauma), and lymphadenopathy. Furthermore, postganglionic etiologies include internal carotid artery dissection, Herpes Zoster infection, and cluster headaches [1,2]. Horner syndrome secondary to osteochondroma is very unusual.

Our literature review found only one other case of Horner syndrome secondary to osteochondroma of the first rib specifically [8]. A case of a 34-year-old female that presented for a regular check-up and was found to have ptosis, miosis, and enophthalmos of the left eye. Additionally, the left eye had a sluggish reaction to light, and on physical exam, a bony mass was visualized and palpated in the supraclavicular fossa. Radiography (x-ray) revealed a mass originating from the posterior third of the left first rib that appeared to be an osteochondroma. The tumor was surgically removed, and the pathology report confirmed the diagnosis of osteochondroma. The patient was followed for eight months after surgery; there was no evidence of recurrence, and her left pupil’s reaction to light improved.

Our case also illustrates the complexity of diagnosing osteochondromas radiographically in a timely manner. Watura et al. encountered a similar problem when they diagnosed Horner syndrome secondary to an osteochondroma of the clavicle in 2015 [3]. They described a case of a 17-year-old female who was admitted for headaches and ptosis. Initially, chest radiography was negative. An MRI revealed a mass arising from the medial clavicle, which confirmed the diagnosis of osteochondroma. This case illustrates a delay in diagnosis as the initial chest x-ray was taken in March 2015, and the actual diagnosis of osteochondroma was not made until May 2015 [3].

Since osteochondromas have been shown to undergo malignant degeneration, it is crucial to make the diagnosis as soon as possible, especially in younger patients. While the delay in diagnosis did not harm the patient in this instance, this may not be the case for every patient presenting with osteochondroma. Not every osteochondroma will warrant a biopsy, but proper imaging to assess for malignant characteristics is important and should not be delayed.

In the present study, a head CT was initially ordered, which was unrevealing, and the patient was discharged. Worsening of symptoms led to the patient being readmitted, where an MRI of the brainstem and cervical spine was ordered along with an MRA of the head and neck. MRI of the cervical spine revealed a mass. Thoracic CT was subsequently ordered, which confirmed the mass and a CT-guided biopsy diagnosed osteochondroma. 

Analysis of this case report may showcase effective imaging modalities that should be utilized in patients presenting with Horner syndrome. Both the current study and Watura et al. were able to best image the mass with a CT of the Thoracic spine. Further studies can include an ultrasound or CT-guided biopsy if the mass demonstrates a thickened cartilaginous cap. Imaging modalities such as chest radiography, CT head, and MRI head/neck did not help locate the mass. The SNS fibers originate in the hypothalamus, but they dive down into the upper cervical and thoracic spinal cord before making their way back up to the head, eye, and neck. Thoracic CT is an important study to consider in a patient presenting with Horner syndrome since lesions anywhere in the upper thorax can impinge on SNS fibers. We believe the lesion in this case report could have been identified earlier if a CT of the Thoracic spine was done at the second ED visit.

## Conclusions

Although it is rare, osteochondroma of the first rib is a cause of Horner syndrome and should be considered in the differential diagnosis. There is a one percent chance that osteochondromas can undergo malignant transformation to chondrosarcoma; thus, it is imperative to identify patients who are afflicted promptly so that they can be triaged for appropriate treatment. Moreover, osteochondromas can be very difficult to image radiographically. Clinicians must consider the path SNS fibers take before innervating structures in the head, eye, and neck. CT of the head and thorax, followed by ultrasound or CT-guided biopsies, are imaging studies that can adequately identify lesions affecting the SNS fibers innervating the aforementioned regions. Compared to CT imaging, other imaging modalities such as chest x-ray and MRI do not articulate the bony anatomy as well.
